# Parliament and the Profession

**Published:** 1919-07-19

**Authors:** 


### Parliament and the Profession.

### Medical Students.

Mr. Fisher, President of the Board of Education, stated that he had been informed by the General Medical Council that the total number of medical students in actual attendance at medical schools in the United Kingdom was 9,490 in January 1919. The first-year students numbered 2,907, as compared with 1,480 in 1913.

### Assistance of the Blind.

Mr. Pratt announced that, pending the legislation that would be necessary before the more important recommendations of the former Departmental Committee could be effectively dealt with, the Government have decided, after full consultation with the Advisory Committee of the Local Government Board, to make grants-in-aid, on an interim basis, of certain services for the blind, to be disbursed on the advice of that Committee.

### State Medical Service.

Major Farquharson asked the President of the Local Government Board whether he is intending to introduce a Bill this Session which would establish a new system of general medical services for the population; and, if so, whether such Bill will provide a whole-time salaried service of doctors, including hospitals and clinics. Dr. Addison replied in the negative, and added that immediately the Ministry of Health Bill becomes law he intends to set up the Consultative Councils under the Bill, including one to advise upon medicine and ancillary services. To these Councils will at once be referred various questions in respect of the development of local health services, and any proposals submitted to the Government will follow the advice given by the Councils.

### Treatment of Syphilis.

Mr. Waterson asked the Minister of Health whether his Department is taking any action in regard to the recent death in St. George's Hospital of a girl who was undergoing treatment for congenital syphilis, and what drug was used in the case. Mr. Waterson also inquired as to similar cases at Cambridge and Dublin. Major Astor replied that the case at St. George's Hospital is under inquiry by the Special Committee appointed by the Medical Research Committee to investigate the results of the treatment of syphilis by salvarsan and its substitutes. He understood that the drug used was novarsenobillon, which is one of the drugs approved by his Department for syphilis, and tested under arrangements made by the Medical Research Committee. There is no sufficient reason for discontinuing the approval of the use of this and similar drugs, and at the Coroner's inquest it was found that the drug was properly administered and in proper amount. There have been several fatal cases following, though not necessarily caused by the administration of these drugs in military hospitals at Cambridge and Dublin during the war, and these cases are also under investigation. The Department has been considering the desirability of requiring special reports direct to the Ministry of all exceptional results following the administration of these drugs.

